# Overcoming the challenges of using automated technologies for public health evidence synthesis

**DOI:** 10.2807/1560-7917.ES.2023.28.45.2300183

**Published:** 2023-11-09

**Authors:** Lucy Hocking, Sarah Parkinson, Avery Adams, Emmanuel Molding Nielsen, Cecilia Ang, Helena de Carvalho Gomes

**Affiliations:** 1 RAND Europe, Cambridge, United Kingdom; 2 European Centre for Disease Prevention and Control (ECDC), Stockholm, Sweden

**Keywords:** Automation, technology, systematic review, evidence synthesis, machine learning, artificial intelligence, public health

## Abstract

Many organisations struggle to keep pace with public health evidence due to the volume of published literature and length of time it takes to conduct literature reviews. New technologies that help automate parts of the evidence synthesis process can help conduct reviews more quickly and efficiently to better provide up-to-date evidence for public health decision making. To date, automated approaches have seldom been used in public health due to significant barriers to their adoption. In this Perspective, we reflect on the findings of a study exploring experiences of adopting automated technologies to conduct evidence reviews within the public health sector. The study, funded by the European Centre for Disease Prevention and Control, consisted of a literature review and qualitative data collection from public health organisations and researchers in the field. We specifically focus on outlining the challenges associated with the adoption of automated approaches and potential solutions and actions that can be taken to mitigate these. We explore these in relation to actions that can be taken by tool developers (e.g. improving tool performance and transparency), public health organisations (e.g. developing staff skills, encouraging collaboration) and funding bodies/the wider research system (e.g. researchers, funding bodies, academic publishers and scholarly journals).

## Background

Reliable, expedient and comprehensive evidence syntheses are a fundamental part of informing public health decision-making, including in crisis situations [1-3]. However, the process of reviewing evidence can be resource and time-intensive [4]. This has been a long-standing problem, and the challenge only grows as the volume of literature expands and the pace of publications increases [5,6]. This became particularly apparent during the COVID-19 pandemic, when evidence was required to be evaluated and synthesised at an unprecedented speed and volume to inform public health decisions [7].

**Figure 1:**
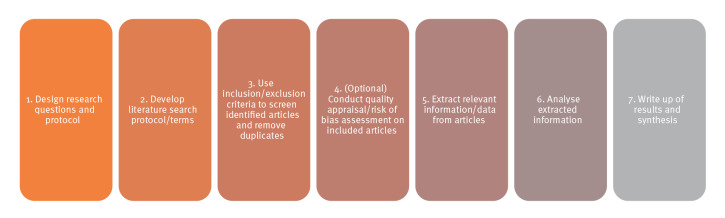
Stages of conducting literature reviews

With the recent development of new technologies for undertaking literature reviews, researchers have been considering how parts of the review process can be completed using automation [6]. Automated tools offer the potential for less time-intensive and more cost-effective reviews without compromising on quality, by supporting key steps of the review process (Figure 1) [8]. However, there are barriers to using automated tools in public health research, and these need to be addressed to ensure the tools are used to their full potential.

This Perspective article is based on learning from a study we conducted exploring the challenges and needs of public health organisations in harnessing the use of automated technologies. The study consisted of a literature review and qualitative data collection from public health organisations and researchers in the field. The methods and findings of this study are described elsewhere in a European Centre for Disease Prevention and Control (ECDC) technical report, written by the authors of this Perspective [9]. The study looked at many different types of technology that have been used to automate parts of the review process, including machine learning technologies, natural language processing, text mining and neural networks (full details of these technologies can be found in the ECDC technical report). Although these technologies can be helpful in automating parts of the review process, the study found that there has been little use of automated literature review tools by public health organisations to date.

This article reflects on the findings of the ECDC technical report to discuss the key challenges faced by stakeholders when using automated literature review tools and actions that could be taken to support public health organisations to make better use of these tools. It first summarises challenges with automated tools followed by suggested main areas of action for three groups: (i) how tool developers can better understand and improve automated systems for public health use; (ii) how public health organisations can use automated tools for evidence synthesis more effectively; and (iii) how funding bodies and the wider research system can support the adoption of automated literature review tools within the research community. While we focus specifically on the adoption of automated technologies within public health, the actions described here could also be applied to other sectors.

## Challenges related to the use of automated tools for evidence synthesis

The most relevant challenges for using automated tools for evidence synthesis in public health that were identified in the study are summarised in the Box.

Box **Challenges for using automated evidence synthesis tools in the public health setting**

- Bias, reproducibility, and performance;
- Trust and transparency;
- Time, skills, and capacity;
- Funding and costs;
- Shared learning and collaboration.

Automated tools for evidence synthesis can be affected by bias. For example, algorithm-based technologies that do not use active learning (i.e. they do not iteratively learn from input or supervision from a human reviewer and instead are trained using training datasets), rely on high-quality training datasets which are created and annotated by skilled researchers. However, datasets used to train algorithms are not always representative and can lead to incomplete or skewed representations of the available evidence in medical databases, which can in turn affect the accuracy and validity of automated evidence synthesis tools. If training data are collected from a single knowledge database or particular epidemiological data source that only reflects the situation in one specific area of the world, the algorithm will recognise the characteristics of that specific data source rather than provide a representation of the real-world situation [10]. This issue is particularly challenging in the context of existing publication bias in medical databases and in research funding towards English-speaking and high-income countries. Where active learning is used, automated tools also reflect the biases of human reviewers. In addition, there is not one ‘silver bullet’ tool that is able to completely automate a review from start to finish. This means users need to combine multiple types of software to complete discrete review stages. For example, one tool may be needed to screen and select studies and a second tool may be needed to support automated data extraction. Often, tools do not integrate with each other, which complicates this process.

Participants in focus groups often discussed issues around trusting evidence produced using automated tools and a lack of methodological transparency [9]. For example, uncertainties regarding the tool’s performance (e.g. objectiveness, accuracy and reproducibility) and a lack of information or transparency about each tool’s limitations and decision points (e.g. when screening articles can hamper their adoption. Publishing reviews conducted using automated methods can also be a challenge for researchers due to the lack of clarity from scholarly journals on the standards that need to be met to publish in them such as if it is acceptable to use automated tools or not, and what needs to be in place for these tools to be considered robust.

While some users of automated evidence synthesis tools reported in the literature that the tools are simple and user-friendly [11], focus group participants from public health organisations in the ECDC-funded study were generally more critical because of the time, skills and capacity required. This would be especially true during the transition phase from traditional, manual to more automated processes. Smaller organisations, such as public health authorities within small countries, may face greater challenges in providing staff with sufficient time to get trained or acquainted with new tools, particularly as staff are likely to have multiple responsibilities and competing priorities. Small organisations may also lack access to adequate IT skills as some tools require specialised skills (e.g. coding) to use. In addition, while evidence synthesis follows a highly structured process as described in Figure 1, and automated tools can reduce the burden, repetitiveness and workload of reviews, these tools do not remove the need for human input and oversight altogether. Resources are needed, sometimes at significant levels, to check the accuracy of results and offer intellectual input that the technology cannot perform, such as in interpreting results [11]. For example, tools may over-include studies when screening, which requires staff time to double check screening classifications. Finally, although automated tools could be useful during crisis periods when evidence is rapidly developing, introducing new ways of working during these times can be difficult due to the additional pressures of competing priorities and lack of time. In addition, concerns were raised over job losses when introducing automated ways of working.

Many tools require upfront purchase of a subscription or license, which may not be feasible for all organisations, particularly if public health budgets are being constrained. While funding bodies may also benefit from the use of automated tools themselves e.g. through faster publication of the research they fund and more cost-effective use of funding, they may not recognise the financial costs of automating a review, especially when introducing this approach for the first time. This may result in funding bodies underestimating the up-front investment required to realise potential future returns.

Organisations working in public health have a lot to gain from collaborating with each other and with evidence synthesis developers and researchers. However, there is currently limited collaboration in practice.

In the remainder of this perspective article, we reflect on the actions that could be taken by tool developers, public health organisations, funding bodies and the wider research system to overcome these challenges.

## Ways for tool developers can support public health organisations to use automated technologies for evidence reviews

**Figure 2:**
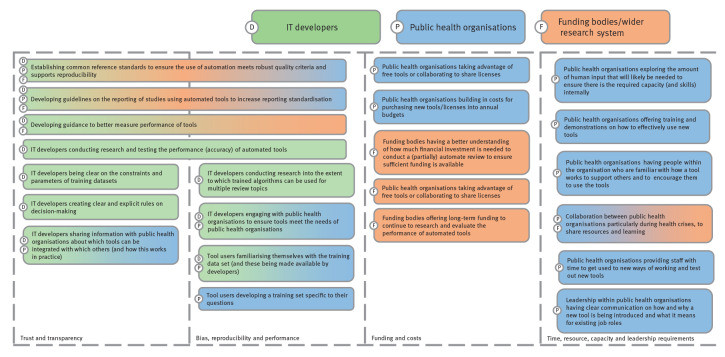
Summary of actions and collaboration needs for tool developers, public health organisations and the wider research system to successfully adopt automated tool systems for evidence reviews

To shift automated tools from being viewed as a ‘black box’ to a ‘glass box’, developers of automated tools should make quality training datasets available (where relevant) and provide clear and sufficient information on how the algorithm makes decisions. However, some technologies which use artificial intelligence may never be completely transparent. Developers should also clarify the limitations of their tools to help users decide whether a particular algorithm or tool is appropriate for their needs and particular question(s), or if they need to develop a new training data set [10]. Applying descriptions and terminology that would allow end users without programming expertise to make decisions about which technology to use and how would be particularly helpful (Figure 2). It is also important to acknowledge different needs across research and technology sectors as researchers are likely to require higher tool accuracy than technology developers are aware of.

Developers must ensure that the training datasets, where used, are of high quality by collecting diverse and representative data, e.g. from high-risk and low-risk populations. Appropriate measures should also be applied to reduce the potential for bias, such as data cleaning and normalisation, choosing appropriate algorithms and evaluating their performance or using fairness metrics to identify remaining biases.

Where tools are designed and trained for specific reviews or topics and made available for wider use, their accuracy should be tested on a wide range of topics. This would help explore the extent to which algorithms trained with datasets in one topic can be reliably used for reviews on others. Developing integrated evidence synthesis workflows can address the current problem of too many highly specialised tools for very discrete review use cases and tasks. This can be supported by tool developers collaborating with public health organisations to better understand their needs and create tools that can be integrated with each other. There are also existing interoperability standards that can be used to improve integration between tools.

## Ways public health organisations can support the use of automated technologies for evidence reviews

It is important that public health organisations allow time for staff to test new approaches and get used to new ways of working, as well as offer regular training and demonstrations on how to use the technology (Figure 2). This requires up-front investment. Having a champion among staff with knowledge of how the tool works embedded in a team, and who works closely with tool developers, can be useful to support colleagues with queries and to act as a mentor. For example (from the focus groups), a European public health authority rolling-out automated tools to review COVID-19 evidence had a ‘super-user’ expert staff who trialled each tool before it was implemented and so became highly knowledgeable on it.

As automated literature review tools still require human input, it is important to have clear and comprehensive communications from leadership about how and why a new tool is being introduced, what it means for existing roles and ways of working and where to go for support. This is important for staff to understand the changes being implemented and feel supported by leadership. Clear communication was used by leaders within a European public health authority when implementing automated tools to rapidly review literature on COVID-19 in an example provided during the focus group discussions. A communication strategy was developed and shared with staff to highlight what changes to expect and how their valuable skills were still required, enabling staff to get on board with the change.

Using free to use tools can help to overcome financial challenges, as can collaborating with other organisations to share licensing agreements where this is permissible.

Greater collaboration between public health organisations and funding bodies/ the wider research system including researchers, funding bodies, publishers and scholarly journals, with regard to conducting evidence syntheses, with or without automated tools, discussed further in the next section, can help to share knowledge and learning.

## Ways funding bodies and the wider research system can support the use of automated technologies for evidence reviews

It is important for funding bodies to allow for short-term funding to cover immediate costs of automating reviews (Figure 2). Long-term funding is also important to continue improving and evaluating tools and to encourage researchers to use automation to a greater extent.

Common reference standards and guidance for measuring the performance of automated technologies and guidance to standardise reporting of automated review approaches (such as through existing reporting guidelines) [12-14] are needed to ensure automated approaches meet quality criteria that allow reviews to be reproduced by other researchers and to support transparent reporting of research. Research funding bodies and scholarly journals can also encourage authors to declare the use of such tools and reflect on how the tool worked in practice, what did and did not work well, to share practical learnings.

Forming public health-specific networks and creating shared repositories of tools including and sharing their advantages and disadvantages, training datasets for different research topics and training activities can support mutual learning. This is an important step in building capacity to improve the evidence synthesis process and the use of evidence for public health decision-making, particularly during crisis situations, through automated tools.

## Conclusion

Collating and reviewing evidence is a vital part of public health decision-making. However, traditional evidence synthesis approaches are no longer sufficient to cope with the large volume of literature. Automated technologies offer public health organisations an opportunity to review evidence in a more efficient way, without compromising on scope. This is particularly important for dealing with public health crises, such as the COVID-19 pandemic during which hundreds of thousands of scientific articles have been published [15]. However, significant barriers to public health organisations looking to adopt new technologies remain. These barriers are related to tool developers’ understanding of public health organisation needs in terms of automated systems, how public health organisations can train their staff to use automated tools more effectively and how funding bodies and the wider research system can support the research community to adopt automated tools. Figure 2 summarises the actions that could be taken across these three groups including where collaboration is key to success. Developing in-house skills in relation to using automated technologies, having dedicated resources and capacity, supportive leadership, increasing cross-sector collaboration and improving tool performance and transparency are just some of the key ways public health organisations can better take advantage of these tools.
